# 3’UTR Shortening Potentiates MicroRNA-Based Repression of Pro-differentiation Genes in Proliferating Human Cells

**DOI:** 10.1371/journal.pgen.1005879

**Published:** 2016-02-23

**Authors:** Yonit Hoffman, Debora Rosa Bublik, Alejandro P. Ugalde, Ran Elkon, Tammy Biniashvili, Reuven Agami, Moshe Oren, Yitzhak Pilpel

**Affiliations:** 1 Department of Molecular Genetics, Weizmann Institute of Science, Rehovot, Israel; 2 Department of Molecular Cell Biology, Weizmann Institute of Science, Rehovot, Israel; 3 Division of Biological Stress Response, The Netherlands Cancer Institute, Amsterdam, The Netherlands; University of Maryland Medical School, UNITED STATES

## Abstract

Most mammalian genes often feature alternative polyadenylation (APA) sites and hence diverse 3’UTR lengths. Proliferating cells were reported to favor APA sites that result in shorter 3’UTRs. One consequence of such shortening is escape of mRNAs from targeting by microRNAs (miRNAs) whose binding sites are eliminated. Such a mechanism might provide proliferation-related genes with an expression gain during normal or cancerous proliferation. Notably, miRNA sites tend to be more active when located near both ends of the 3’UTR compared to those located more centrally. Accordingly, miRNA sites located near the center of the full 3’UTR might become more active upon 3'UTR shortening. To address this conjecture we performed 3' sequencing to determine the 3' ends of all human UTRs in several cell lines. Remarkably, we found that conserved miRNA binding sites are preferentially enriched immediately upstream to APA sites, and this enrichment is more prominent in pro-differentiation/anti-proliferative genes. Binding sites of the miR17-92 cluster, upregulated in rapidly proliferating cells, are particularly enriched just upstream to APA sites, presumably conferring stronger inhibitory activity upon shortening. Thus 3’UTR shortening appears not only to enable escape from inhibition of growth promoting genes but also to potentiate repression of anti-proliferative genes.

## Author Summary

MicroRNAs (miRNAs) are regulators of gene expression. Typically they recognize a binding site in genes' sequences and exert a repressive effect. This scheme prescribes a regulatory network that determines which gene is regulated by which miRNA. Yet this is a static sequence-based scheme that might not support dynamic changes in network wiring. Can genes become subject to, or be released, from the regulation of a miRNA in a manner that depends on the physiological state of cells? Here we describe such a dynamic mechanism. It is established that miRNA regulation is often more effective when their binding sites reside near the end of the target mRNA or right after the coding sequence STOP codon. Thus, a site distant from the mRNA’s end might be latent as it will not bind efficiently its corresponding miRNA. Yet, in particular physiological states, e.g. cancer or rapidly proliferating cells, mRNA ends tend to become shortened. So far it was suggested that such shortening may serve to release proliferation-favoring genes from miRNA repression by eliminating their binding sites from the mRNA. We propose a mirror image, complementary mechanism that acts upon genes that need to be repressed during proliferation. Specifically, we propose that mRNA shortening can dynamically activate latent repressive miRNA binding sites by bringing the mRNA end close to them. We mapped the ends of all mRNAs in proliferating cells and found that cancer-enriched ends are strikingly positioned closely downstream to a high density of potentially latent binding sites, which are retained in the short mRNA but are now close to the new ends. This may enable such potentially latent miRNA sites to become dynamically activated upon proliferation. Remarkably, this mechanism targets preferentially pro-differentiation and antiproliferative genes, which are often repressed in cancer.

## Introduction

RNA polyadenylation is a pivotal molecular process, which plays important roles in ensuring the stability, nuclear export and efficient translation of mRNA. Cleavage of the mRNA in its 3’ untranslated region (UTR), prior to addition of the poly(A) tail, is instructed by a polyadenylation signal (PAS) [1]. In fact, most genes contain multiple PASs with different affinities to the cleavage machinery, resulting often in alternative polyadenylation (APA) sites. Typically, the distal PAS, which creates the longest 3’UTR, contains the canonical signal (AAUAAA), while alternative sites more often contain variations on that signal; as such, the distal site is usually more conserved than the alternative PAS and is more frequently used [2].

While the usage pattern of APA sites is highly conserved, specific cell types and conditions appear to be more prone than others to increased use of APA [2]. One example is that of cell proliferation, where a shift towards increased APA site usage has been observed, leading to shortening of many 3’UTRs [1,3]. Likewise, the transition from differentiated cells to induced pluripotent stem cells is also accompanied by global 3’UTR shortening [4]. A tendency to use proximal APA sites is seen also in particular tissues, such as placenta, ovaries and blood [5]. In contrast, processes such as embryonic development and myogenic differentiation of cultured myoblasts are accompanied by progressive lengthening of 3’UTRs [1]. Notably, cancer cells have been reported to exhibit even more extensive 3’UTR shortening than non-cancerous proliferating cells [6].

microRNAs (miRNAs) are small non-coding RNAs that regulate the translation and stability of their target mRNAs. They recognize such mRNAs by one or several motif site sequences, often located within the 3'UTR of the mRNA, which are complementary to bases 2–8 (called "seed") in the 5' end of the mature miRNA [7].

**Fig 1:**
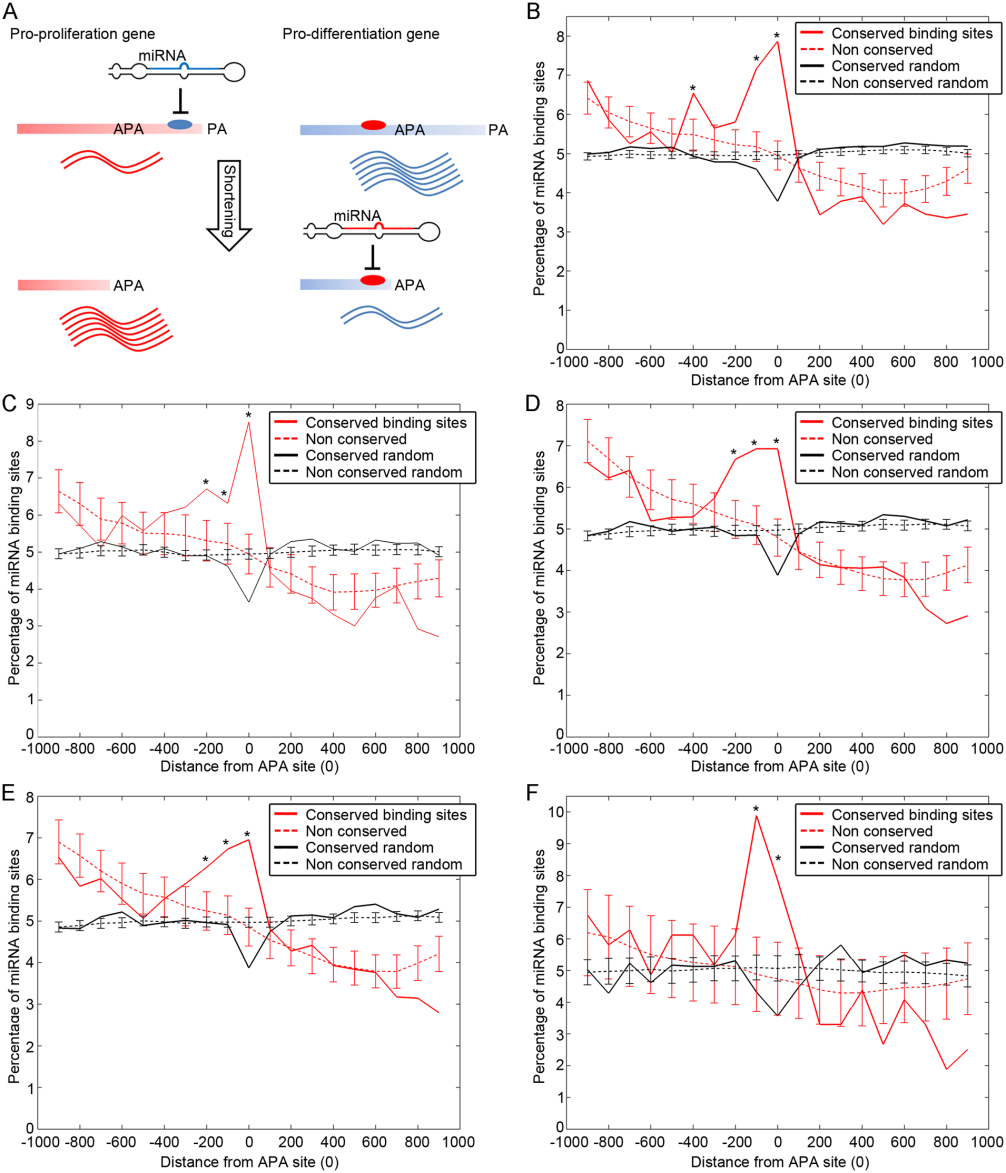
Conserved miRNA binding sites are enriched immediately 5' to APA sites. **(A)** A pro-proliferation and a pro-differentiation genes are shown, each being subject to a different miRNA. The pro-proliferation gene contains the miRNA binding site close to the full-length 3’ UTR (near the polyadenylation (PA) site, while the pro-differentiation gene contains its site in the middle of the full-length 3’ UTR, yet immediately upstream to an APA site. The left side represents a differentiated lowly proliferative tissue, in which both genes mainly feature the long UTR. Under this condition the pro-proliferation gene is repressed while the pro-differentiation gene is free from repression. On the right side the cells are proliferative and the 3’ UTRs of the two genes are shortened. In this condition the pro-proliferation gene is relieved from repression since the binding site no longer exists in its UTR. In contrast, the pro-differentiation gene now becomes subject to increased repression since its binding site now becomes closer to the new UTR end. **(B-F)** Conserved and non-conserved miRNA binding sites around the APA site (point 0), for all genes with at least 1000 bases of 3’ UTR sequence from each side of the APA site, in different cell lines: WI38 (B), U2OS (C), BJ (D), MCF10A (E) and Mouse Muscle Tissue (Genes with at least 5000 bases 3’ UTR sequences from each side of the APA site) (F). * indicates p-value<0.05.

An interesting aspect of 3’UTR shortening is the interplay with miRNAs. If the binding site of a particular miRNA resides within the part of the 3’UTR that is removed upon shortening, typically when an APA site is used, regulation of the target mRNA by this miRNA is abrogated (Fig 1A). Sandberg et al. [3] and Mayr and Bartel [6] reported that in proliferating or cancerous cells, some APA isoforms of proto-oncogenes tend to be more stable, generate more protein and promote higher neoplastic transformation rates. This was found to be due to their ability to escape miRNA regulation, as the corresponding binding sites were eliminated in the shorter 3’UTRs [3,6]. The notion of escaping miRNA regulation via APA is also supported by additional examples, e.g. *ABCG2* that escapes regulation by miR-519c by 3’UTR shortening in drug resistant cells [8], and Hsp70, which is alternatively polyadenylated upon ischemia or heat shock and thereby escapes miR-378* regulation [9].

A typical 3’UTR comprises many potential miRNA sites; however, only a small fraction of those are functional in any given cell type and state. The position of the site along the 3'UTR can influence its functionality. In particular, conserved functional miRNA binding sites tend to be positioned near the beginning and end of the 3'UTR [10]. Nonetheless, potentially functional sites might still reside closer to the middle of the full length (FL) 3’UTR. When an APA site is used, the distal part of the FL 3’ UTR is eliminated, together with the functional miRNA binding sites residing within it. However, the region immediately upstream to the APA site, previously located away from either ends of the 3’UTR, now becomes positioned close to the new 3’ end. This can potentially render miRNA sites located in that region more functional for miRNA-mediated repression (Fig 1A). In this manner, APA may augment the functionality of potential miRNA binding sites residing 5' to the APA site. A recent study indeed supports this conjecture, in a cell type-specific manner. Nam et al. [11] compared transcripts with different 3’UTR lengths in HEK293 and HeLa cells, and found that in the cells where the miRNA binding site was closer to the 3’ end of the transcript (due to the different in 3’UTR lengths), repression by miRNAs was stronger [11]. We now report that this is a global phenomenon, where 3’ UTRs in diverse cell lines are strategically shortened precisely downstream to conserved miRNA binding sites; this occurs particularly in differentiation-related genes, and involves preferentially targets of miRNAs that are induced in proliferation and cancer. 3’ UTR shortening may also serve as a dynamic tool to functionalize otherwise latent, binding sites residing more proximally.

## Results

### Conserved miRNA binding sites are enriched immediately 5' to APA sites

In order for an APA event to potentiate targeting by miRNAs, potentially functional sites should exist 5' to the APA site, at a distance from the APA that is comparable to that typically seen between conserved miRNA sites and the canonical 3’ UTR ends. We have previously shown this region to be ~250 nucleotides from either end of the 3'UTR [12].

To identify transcriptome-wide APA sites, we subjected WI-38 human embryonic lung fibroblasts and their immortalized derivatives obtained through sequential serial transfers towards increased proliferation and transformation [13,14] to 3' sequencing and analysis [15]. The analysis included primary fibroblasts (“Control”), slow growers (early passage after immortalization), fast growers (extensive passaging after immortalization) and fast growers transformed by constitutively activated mutant H-RasV12 (“Ras”). 5765 genes were found to have at least one APA site in at least one of the cell types in this experimental system. Somewhat unexpectedly, we did not detect significant differences in the overall extent of global shortening between the different cell types. Remarkably though, when we aligned all genes with at least one APA event according to their most proximal APA site, a significant enrichment of conserved binding sites was observed within the 300 bases immediately upstream to the APA site (Fig 1B for control cells, S1A Fig for all time points). A similar picture emerged from the analysis of previously published 3'seq data [15,16], obtained in different cell lines (Fig 1C–1E). Of note, in those earlier experiments, APA was compared in MCF10A and BJ cells under growth arrest vs. proliferation and transformation, revealing significant shortening in the proliferating cells. Our inability to detect comparable shortening on a genome-wide scale in the WI-38 analysis may therefore stem from the fact that all compared states involved proliferating cells; alternatively, this may reflect the embryonic origin of WI-38 cells. Notwithstanding, though we do not see a genome wide tendency for shortening in these samples, we do observe many genes that do feature a shortened version, and we analyze them below.

A trivial reason for the similar enrichment of conserved miRNA sites upstream to APA sites in the different datasets could be that in all datasets the same genes undergo the same APA events. However, this pattern was still retained also when we performed a similar analysis only on APA-positive genes that differ between pairs of cell lines (examples in S1B–S1E Fig). Moreover, analysis of 3'seq data from mouse muscle tissue [16] revealed a similar peak of conserved miRNA binding sites upstream to the APA sites (Fig 1F). In the mouse tissue fewer genes were found to undergo APA, an observation which might reflect the highly differentiated state of the cells. Hence, in multiple cell types and in two mammals, distinct APA sites reside preferentially closely downstream to conserved miRNA binding sites, effectively repositioning such centrally-located miRNA binding sites and placing them in proximity to the 3’ end of the shortened transcript. In support of the emerging notion, when we compared the distribution of conserved miRNA binding sites between genes possessing at least one APA site in WI-38 cells and those without APA sites, we found (S2 Fig) that genes with APA sites tend to harbor fewer conserved binding sites near the distal end of their full length 3'UTR, relative to those without an APA (p-value = 6.5e-279, Student T-test). Conversely, genes with APA sites are relatively enriched in conserved miRNA binding sites within the proximal half of their 3’UTR (S2 Fig). This observation is intriguing: if APA merely serves to eliminate miRNA binding sites residing near the 3' end of the full length mRNA, one would expect APA-positive genes to be more enriched for functional miRNA binding sites near that end, providing them with an efficient on/off switch controlled by APA. The fact that the opposite trend is actually observed strongly suggests that the interplay between APA and miRNAs may allow regulation that is richer than mere binding sites elimination. Specifically, this may serve as further indication that the shorter 3’ end, positioned immediately upstream to the APA site, can dynamically potentiate new miRNA binding sites as they become positioned closer to the 3’ end of the shorter transcript.

### miRNA binding sites located 5’ to APA sites are probably selected for miRNA targeting

**Fig 2:**
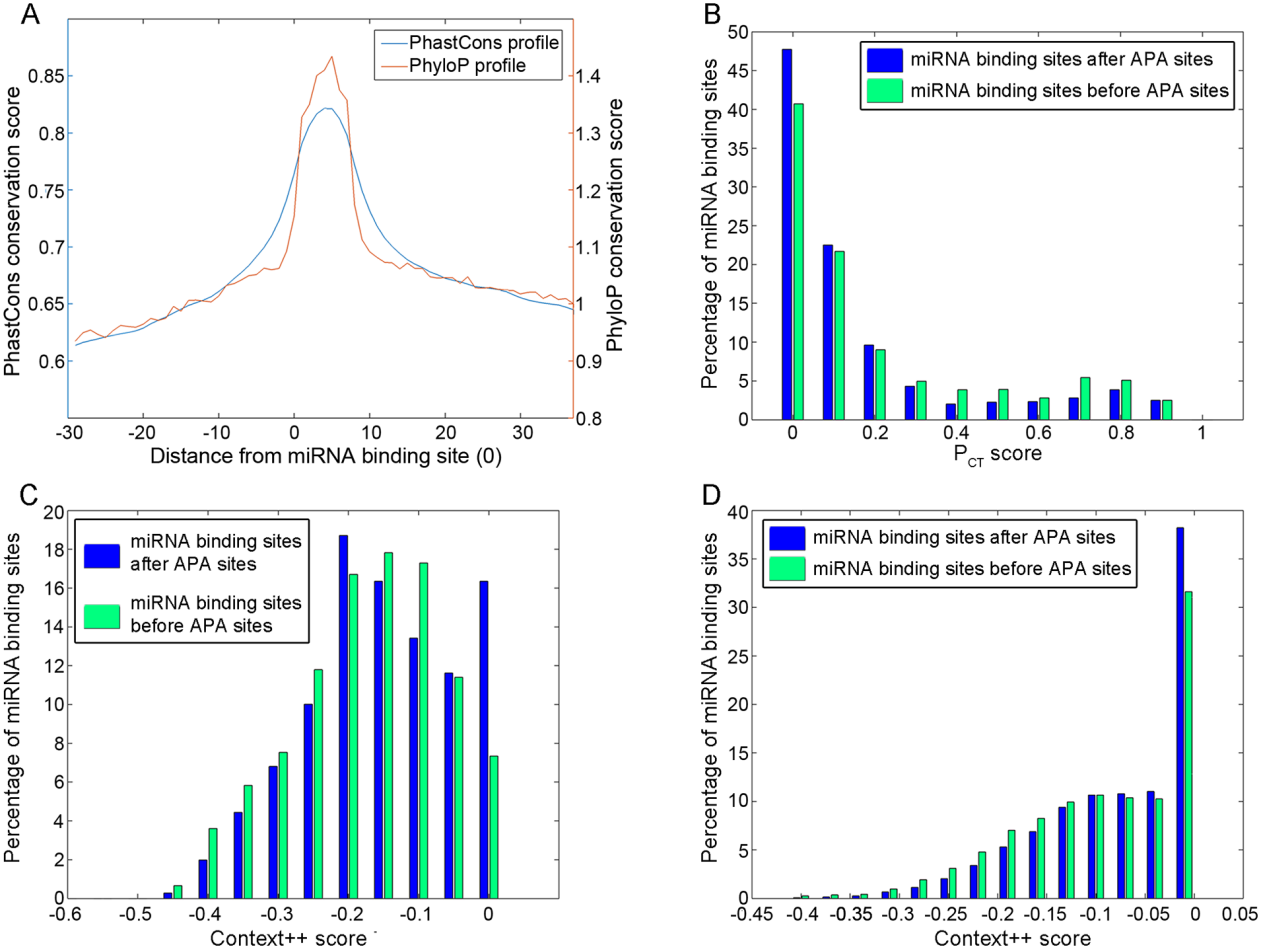
miRNA binding sites located 5’ to APA sites are probably selected for miRNA targeting. **(A)** Conservation profile of 30 bases around conserved miRNA binding sites which are located in the 300 bases 5’ to APA sites. Two conservation scoring systems are displayed—PhastCons and PyhloP. **(B,C,D)** P_CT_ conservation scores (B) and Context++ scores (C,D) of miRNA binding sites located 300 bases 5’ or 3’ (before and after) APA sites, for genes with at least 500 nucleotides from each side of the APA site. For the context++ scores the miRNA binding sites are divided to conserved (C) and non-conserved (D).

Analysis of the conservation state of each miRNA binding site by itself is an accepted indication for its functionality [10,17,18]. However, such conservation might be due to other attributes, for example another functional feature of the 3’UTR residing in this location. We therefore asked whether the high conservation of the miRNA sites located upstream to APA sites can be attributed to conservation of their neighborhood, or is preferentially targeting the miRNA sites? To address this question we looked at the conservation profile around APA sites, for genes with and without conserved miRNA binding sites in the 300 bases 5’ to the APA sites. As can be seen in S3 Fig, the profile of the genes without conserved sites 5’ to APA sites is lower specifically in this area, indicating that the high conservation present for the other genes might be due to the presence of miRNA targeting, and not merely APA sites. We also looked at the conservation profile of all conserved miRNA binding sites located in the 300 bases 5’ to APA sites. For that we used PhastCons and PyhloP [19,20] as provided by the UCSC browser. We observed a sharp peak of conservation exactly overlapping the 7 bases of the binding sites, whereas its surroundings are significantly less conserved (Fig 2A), strongly arguing in favor of a selective pressure to conserve specifically the conserved miRNA binding sites. Another measurement of site conservation is the P_CT_ score, which controls for the 3’UTR surroundings, dinucleotide conservation and other parameters unrelated to miRNA functionality [21]. Importantly, this score allows to assess the extent to which conservation of a site is likely to be due to miRNA functionality. We compared the distribution of P_CT_ scores for miRNA binding sites 300 bases 5’ and 3’ to APA sites, and found that the scores are significantly higher for the sites located just 5’ to the APA sits (Fig 2B, p-value = 2e-7, Student T-test). Moreover, we employed the Context++ scoring system of miRNA binding sites, which takes into account many additional parameters of each site and its surroundings beyond mere conservation, and provides a score for the probability that this site is indeed functional [22]. We compared the scores of the binding sites 300 bases 5’ and 3’ to APA sites, for conserved and non-conserved sites. For both groups, the scores for sites located 5’ to APA sites were significantly lower (hence indicating higher functionality) (Fig 2C and 2D, p-value = 2e-6, 7e-165, Student T-test). Together with the P_CT_ score analysis, this argues that these sites are more likely to be indeed functional.

In sum, the above analyses strongly suggest that the miRNA binding sites located just 5’ to APA sites are functional beyond sequence conservation. Since the Context++ scores of non-conserved miRNA binding sites were also better for the ones that are positioned just 5’ to APA sites, we can conclude that even the non-conserved binding sites are probably more functional for miRNA targeting when located in that region.

### Embryonic pattern specification genes and targets of the proliferation miRNA cluster are enriched for conserved sites immediately upstream to APA sites

**Fig 3:**
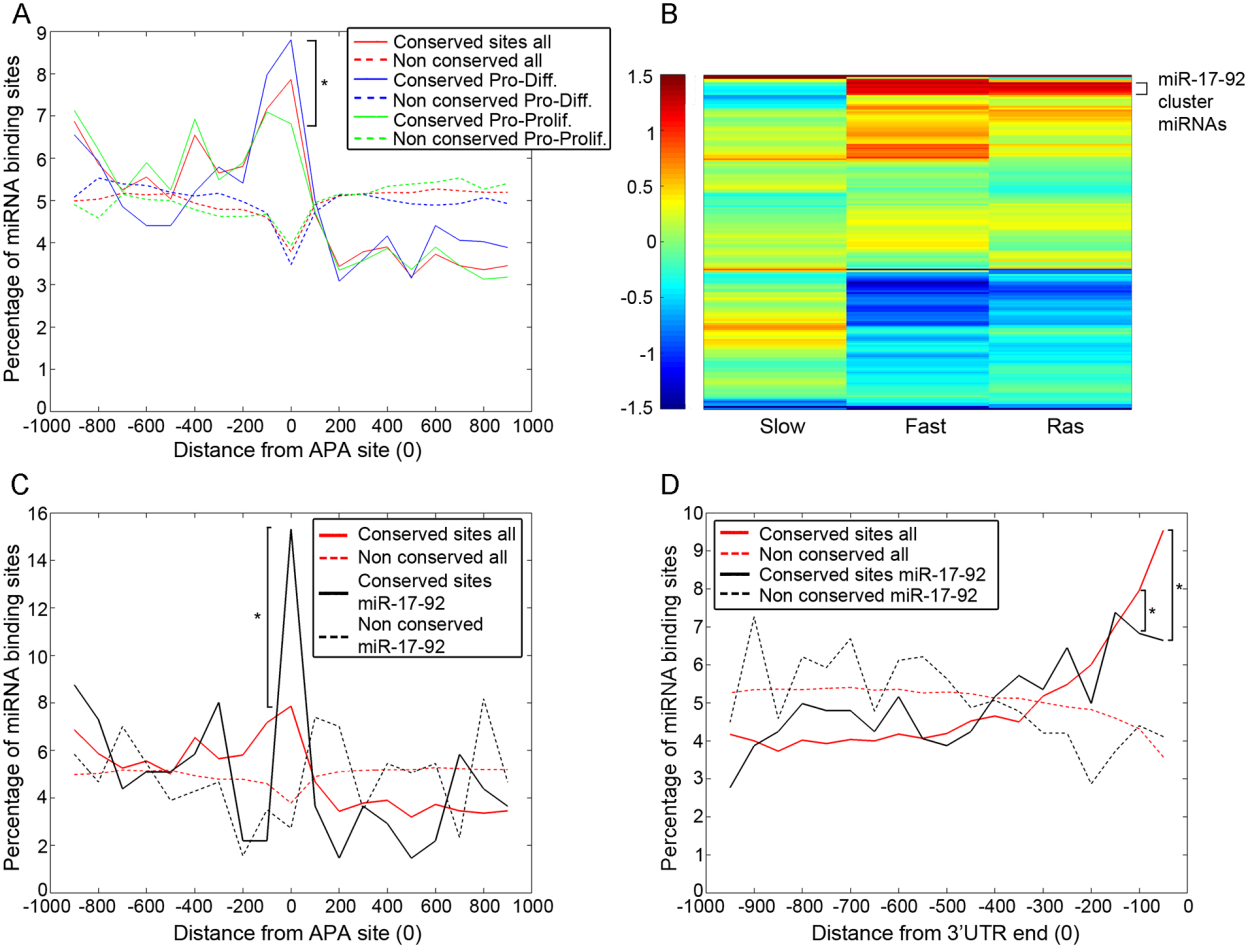
miRNAs and genes enriched for conserved binding sites upstream to APA sites. **(A)** Conserved and non-conserved miRNA binding sites for genes with APA site and at least 1000 3’ UTR bases around it are divided in different groups according to codon usage correlation. **(B)** Heat map representing the mean fold change (log2 scale) of each miRNA in the miRNA array experiment from the control (primary cells) sample. **(C)** Conserved binding sites around the APA site for miRNAs in the miR-17-92 cluster and for all miRNAs. Only genes with at least 1000 3’ UTR bases before and after the APA site were considered for the analysis. **(D)** Conserved binding sites upstream the long 3’UTR end for miRNAs in the miR-17-92 cluster and for all miRNAs. Only genes with at least 1000 bases 5’ to the long 3’UTR and with APA site were considered for the analysis. * indicates p-value<0.05 for the difference between conserved miRNA binding sites between Pro-Diff. and Pro-Prolif. Genes (A), or between all miRNAs and miRNAs from the miR-17-92 cluster (C,D).

In non-proliferating cells, most genes express mainly the full length version of their 3’UTR. However, in proliferating cells and particularly in cancer, increased usage of APA sites has been shown to enable miRNA binding sites elimination from the 3’UTRs of proliferation-associated genes [1,6]. By the same rationale, we predicted that if our above observations are physiologically relevant, then genes becoming more susceptible to miRNA regulation (and hence more effectively repressed) owing to APA would tend to be those that should be preferentially downregulated during proliferation and in cancer. To address this prediction, we initially compared two gene sets: those at the core of the cell cycle machinery, and those involved in patterning of the embryo during development. We chose these two gene sets as they represent two opposing classes of archetypical proliferation and differentiation genes [23]. Yet, these gene sets are relatively small and for only a portion of them we detected APA events. We therefore sought to expand these gene sets to include functionally related genes and thus gain further statistical power. To that end, we expanded each gene set to include additional genes either by similar codon usage (see M&M) or based on the GSEA tool [24]. We then analyzed the miRNA binding site landscape around the APA sites of the two gene sets. Remarkably, we observed a significant difference between the two groups of genes: while the proliferation-related genes (“Pro-Prolif.”) display only modest enrichment of conserved miRNA binding sites immediately upstream to the APA site, the differentiation-related genes (“Pro-Diff.”) show a markedly elevated abundance of conserved miRNA binding sites in the corresponding region (Fig 3A and S4A–S4C Fig). This strongly suggests that differentiation-related genes are more prone than proliferation-related genes to regulation by miRNA binding site potentiation via APA. This is in line with the documented increased APA usage during proliferation and cancer, when differentiation-related genes are expected to be downregulated.

These results suggest that, in addition to its documented ability to alleviate miRNA-mediated repression of proliferation genes, 3’ UTR shortening is also used to potentiate preferentially the repression of differentiation genes.

### Binding sites of a pro-proliferation miRNA cluster may be potentiated by APA

To obtain clues about the miRNAs that bind sites potentiated by APA, we performed comparative miRNA microarray analysis on WI-38 cells and their progressively transformed derivatives [13,14]. We found that most of the miRNAs upregulated particularly in the highly proliferative stages (fast growers and Ras-transformed) belong to the miR-17-92 cluster (Fig 3B and S5 Fig). Indeed miR-17-92 is a well-studied proliferation-associated cluster [25], which includes 6 miRNAs with 4 different binding site sequences. Target genes of these miRNAs are significantly enriched for regulation of differentiation and negative regulation of proliferation (S1 Table), consistent with the notion that these miRNAs promote cell proliferation. Notably, in comparison to all miRNAs on the array, conserved sites for members of the miR-17-92 cluster are significantly enriched immediately 5' to APA sites (Fig 3C). Furthermore, while for all miRNAs in the genome we see a marked increase in the abundance of conserved binding sites near the 3' end of the full length 3’UTR, conserved binding sites of the miR-17-92 cluster are relatively less enriched in that region (Fig 3D). Thus, as compared to the bulk of the cellular miRNAs, miR-17-92 cluster members preferentially bind sites that are located upstream to APA sites rather than near the distal end of the full length 3’UTR. APA is therefore expected to preferentially potentiate the repressive effects of those proliferation-associated miRNAs.

Overall, the above findings further support the conjecture that while APA enables proliferation-associated genes to escape miRNA regulation, it confers increased regulation upon pro-differentiation genes.

## Discussion

It has long been known that APA eliminates functional miRNA sites by truncating the 3’UTR [3,6]. Our present study provides evidence that alternative polyadenylation can also functionalize miRNA binding sites positioned upstream to the APA site. The position of the miRNA binding site along the 3’UTR greatly affects its functionality [10]; we now show that when the RNA is shortened by APA, a new subset of conserved potential miRNA binding site is placed close to the new 3’ end. Presumably, this enables those sites to become more functional (Fig 1A). Previously, Nam et al. concluded that binding sites that are brought closer to the end of the 3’UTR become more functional [11]. We now extend this conclusion and put it within a novel genome-wide dynamic regulatory program. Moreover, we show that this proposed mechanism is potentially of broad relevance, affecting a large number of genes across many tissues and cell types. In our present study, functionality of miRNA binding sites was inferred indirectly by several computational methods. Although the conclusions from the different methods are highly concordant and strongly support our conclusions, it will be of further value to perform directed experiments, monitoring the functionality of those sites and their preferential engagement within the context of the shorter transcript, through the use of methods such as CLIP analysis.

APA enables proto-oncogenes to escape miRNA regulation by eliminating binding sites located towards the distal end of the full length 3’UTR [3,6]. Complementing the picture, our study shows that anti-proliferative genes can also be modulated by APA, but in an opposite direction: they may gain functional binding sites and become more susceptible to repression. This is not to say that only anti-proliferative genes gain functional sites as a consequence of APA events; reality is likely more complicated, and it is entirely possible that other groups of genes would also gain functional miRNA binding sites by a similar mechanism. However we do see a significant enrichment for conserved binding sites prior to APA sites in genes with pro-differentiation characteristics, which might indicate that these genes will be more significantly sensitized to miRNAs when global shortening occurs. As in all other aspects of biology, context matters. Hence, such sensitization becomes more meaningful under conditions where the pertinent miRNAs, targeting the functionalized sites within the shortened 3’UTR, are also upregulated. Indeed, this is what we see in the case of the miR-17-92 cluster, which is upregulated in highly proliferative cells. Notably, significant enrichment of binding sites was seen specifically for miRNAs upregulated in the proliferating state, rather than for all expressed miRNAs. It is conceivable that, in other contexts, a similar APA-mediated mechanism may serve to selectively render other groups of genes more responsive to repression by the miRNAs that are specifically upregulated in those particular contexts.

**Fig 4:**
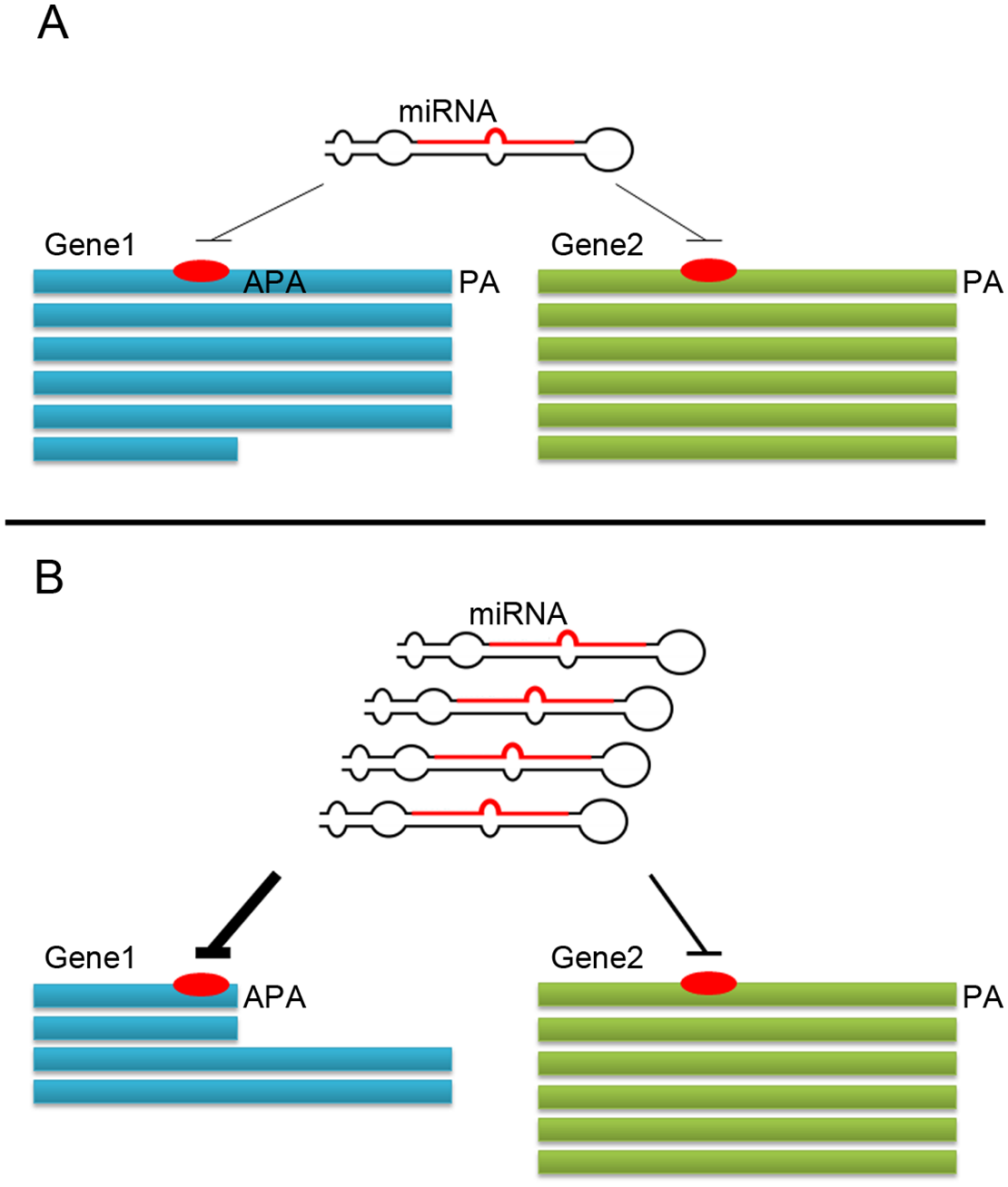
miRNA different regulation between genes according to 3’UTR shortening. Two genes, Gene 1 and Gene 2 contain a binding site for same miRNA and are hence potentially subject to its regulation. In one physiological condition **(A)** the two genes feature mostly the long 3’ UTR, and as the binding site is close to its center, the miRNA can exert little to none of its regulatory effect on the two genes. Upon switch to the second condition **(B)**, Expression of the miRNA is induced. In that condition, Gene 1 undergoes 3’ UTR shortening and its binding site now becomes closer to the UTR’s end, while Gene 2 remains unmodified. Hence, Gene 1, but not Gene 2, now becomes fully accessible to repression by the induced miRNA, and the levels of its short transcript, although upregulated by the shortening, remain low because of the effective targeting of the miRNA. In this way selective 3’ UTR shortening may serve as a dynamic means to differentiate between different targets of the same miRNA, providing the network with additional regulatory flexibility.

What could be the logic behind this dynamic mode of regulation? Consider a miRNA that was induced at a particular physiological condition. If all targets of the miRNA are equally accessible to it that would all be equally affected. If, on the other hand, some of the targets harbor the binding site close to the 3’ UTR’s 3’ end while others contain the sites closer to the UTR’s center, only the first subset of targets would be affected. The second subset could at once become functional targets upon APA and UTR shortening (Fig 4). This mechanism may thus acquire an additional mode of control to the miRNA regulatory network.

## Materials and Methods

### Ethics statement

Low passage WI-38 cells were obtained from ATCC (CCL-75). Slow growing hTERT-immortalized WI-38 cells and their fast growing derivatives, generated by extended passaging in culture of the slow growing cells [13], were kindly provided by Varda Rotter. Ras-Transformed derivatives of the fast growing WI-38 cells were obtained by infection with a recombinant retrovirus expressing H-RasV12 as described in [14].

### 3’Seq and miRNA array

WI-38 cells were grown in 37° in MEM supplemented with 10% non-heat-inactivated fetal bovine serum (Sigma), pen-strep, sodium pyruvate, L-glutamine solution (Beit HaEmek). RNA was extracted using Nucleospin miRNA kit (Macherey-Nagel), according to manufacturer’s instructions.

miRNA array analysis was done in duplicates, using the miRNA Complete Labeling and Hyb Kit (Agilent, 5190–0456) according to the manufacturer's instructions. Briefly, for each sample 100ng RNA was dephosphorylated, denatured, labeled with Cyanine 3-pCp and purified using Micro Bio-Spin 6 Columns. Hybridization was done for 20h with Agilent SurePrint G3 Unrestricted miRNA 8x60K (Release 19.0) arrays. Arrays were scanned using an Agilent DNA microarray scanner, and analyzed using the AgiMicroRna package in R [26] with the RMA algorithm. The heat map was generated with Matlab Clustergram function for the mean fold change of each sample vs. control (mean of the duplicate arrays).

The 3’Seq protocol is based on Jenal et al. [16] and incorporates additional modifications described in Martin et al., 2012[27]. Basically, 25 μg of total RNA were heat-fragmented for 12 minutes in 1x Fragmentation Buffer (Ambion) at 70°C to generate RNA fragments of ~100 nucleotides. Next, the 3’end poly(A) RNA fragments were selected using the Oligotex mRNA Kit (QIAGEN) and RNA was end-repair with T4 polynucleotide kinase for 45 minutes at 37°C following manufacturer’s instructions. Afterwards, RNA 3’ends were blocked for ligation by incubation with 1mM Cordycepin 5′-triphosphate (Sigma) and 10U of polyA polymerase (PAP, NEB), in 1x PAP buffer for 30 minutes at 37°C. Finally, a P7 RNA adapter (5’-CAAGCAGAAGACGGCAUACGAGAU-3’) was ligated to the 5’end using 2U of T4 RNA ligase I and 2.5uM of RNA adapter, for 4h at room temperature. Between each step, RNA was purified using Agencourt RNAClean XP magnetic beads (Beckman Coulter) following the manufacturer’s instructions. At this point, RNA fragments were converted to cDNA employing the Superscript III RT kit (Life Technologies) and an anchored oligo(d)T stem loop primer containing a barcoded Illumina adaptor as in Martin et al (See S3 Table). Next, cDNA was purified twice with Agentcourt AMPure XP magnetic beads (Beckman Coulter) using a ratio 1.5:1 beads:sample. To generate the final 3’Seq library, the cDNA with the correct adaptor sequences was enriched/amplified using Phusion DNA polymerase (Life Technologies) and primers P7 and Illumina_Truseq, for 12 cycles following manufacture’s recommendations. Finally, the 3’seq library was size selected with AMPure XP magnetic beads by two rounds of purification with a ratio 1:1 beads:sample, before being sequenced on an Illumina HiSeq2000 system.

In our protocol of profiling transcript 3' ends, sequenced reads start with a barcode for sample multiplexing which is followed by six Ts whose end marks the precise location where the poly(A) tail starts. These six Ts therefore allow the mapping of poly(A) cleavage sites (CSs) with a nucleotide resolution. After trimming the barcode and six Ts, reads were aligned to the human genome (hg19) using TopHat [28]. Up to two mismatches were allowed in the reads’ seed region (the first 28 nt). As CS location often fluctuates around a major site, we merged reads from all samples and identified "read runs” (that is, genomic intervals that are "tiled" by multiple reads where the distance between the start of consecutive ones is below 10 nt). We considered the local maxima of these runs as the CS locations. We required a spacing of at least 50 nt between consecutive CSs. (In case of lower spacing between CSs, the one supported by a higher number of reads was chosen). Only CSs supported by at least 10 reads (at the location of the CS run maximum) were considered in subsequent analyses. The median length of the runs was 11 nt. Overall, 41,972 CSs were detected in our dataset. Priming of the oligo-dT primer to genomic regions that are A-rich (“internal priming”) could lead to false call of CSs. To reduce the rate of such false calls we extracted genomic sequences of 50 nt centered at the location of the putative CSs, and filtered out CSs that contained in that region a stretch of 10 nt of which at least 8 were As and the rest were Gs. 4,307 suspected CSs were filtered out.

### Prediction of miRNA targets and conservation analysis

miRNA binding sites were defined as perfect 7-mers, which are reverse complement to the seed of the miRNAs, for all human and mouse miRNAs listed in miRBase release 17 [29]. Conserved binding sites were taken from TargetScan release 6.2 [18].

### P_CT_ and context++ scores

P_CT_ scores of all miRNA binding sites of type “7mer-m8” (perfect 7mer) for the conserved miRNA families were taken from TargetScan release 6.2 [18]. Context++ scores of all miRNA binding sites of type “7mer-m8” were taken from TargetScan release 7 [22]. Only miRNA binding sites from genes with at least 500 bases around the APA sites were taken into the analysis.

### Analysis of genes with APA sites

All analyses were done only for genes whose accession number was included in TargetScan 3’UTRs list (as defined in their website). A cleavage site was assigned to a gene if it was included in the coordinates of its 3’UTR (and 20 bases further, after the 3’ end of the 3’UTR). A gene was considered to have an APA site only if it has at least two cleavage sites assigned to its 3’UTR. In all analyses, the APA site that we took into account was the 5’ most in the 3’UTR.

To compute the statistical significance of the main signal—enrichment of conserved miRNA binding sites at particular distances from the APA site, we created a randomized null model. In this null model each binding site’s location was recorded and for each gene a random position of an APA site was drawn from the full length 3’ UTR (omitting the first and last 1000 bases in order to allow inspection of that vicinity around the randomly chosen location). The distance from the binding site and the randomized APA location was computed and the procedure was repeated 10,000 times. This yielded a distribution of distances as shown in all plots, once for conserved binding sites, and once for non-conserved binding sites. * indicates p-value < 0.05 for the null hypothesis that for a specific distance from the APA site, the number of conserved binding sites is similar as in a random APA site, or higher.

### Conservation profiles around APA sites and conserved miRNA binding sites

PhastCons and PhyloP scores of each base in the genome (hg19) were taken from UCSC [19,20]. The profile around APA sites of genes was for genes with at least 1000 bases from each side of the APA sites. The profile around conserved miRNA binding sites was for all miRNA binding sites located in the 300 bases 5’ to the APA site. The sites were aligned and the mean conservation profile was calculated.

### Codon usage, Gene Set Enrichment Analysis (GSEA) and miRNAs analysis

We defined the “Pro-Proliferation” and “Pro-Differentiation” gene sets as follows: We began with the Gene Ontology sets termed “M-phase of cell cycle” and “Pattern Specification”, two gene sets that were recently shown [23] to serve as archetypical proliferation and differentiation genes, with distinct codon usage. To augment the number of genes belonging to each of the two sets we searched the entire genome for additional genes whose codon usage was highly similar to either of the two groups, thus expending the two sets from 92 and 82 genes originally to 229 and 136 (For correlation threshold 0.7: 226 and 147, for correlation threshold 0.8: 172 and 125). We expanded the two original gene sets (“M-phase of cell cycle” and “Pattern Specification”) also by correlation to other GO groups. These groups were taken as the top 50 groups for each original group using the GSEA tool track C5 [24].

After computing the miRNA binding site distribution around APA sites for the genes in each of the sets we estimated a p-value on the difference between conserved miRNA binding sites density at each distance from APA sites as follows: we repeated 10,000 on randomly partitioning the genes with high correlation to the two groups into two groups, one with 229 genes and one with 136 genes for the codon usage expansion threshold 0.75 correlation, and 175 and 167 genes in the expansion by the GSEA tool. In each such random partition we recorded, at each location relative to the APA, the fraction of conserved miRNA binding sites. The p-value was estimated as the fraction out of the 10,000 repetitions in which the real partition into “Pro-Proliferation” and “Pro-Differentiation” resulted in a difference in binding sites count. * indicates one-sided p-value < 0.05 for the null hypothesis that for two groups of these sizes, the difference in number of conserved binding sites is as for the two original groups or higher.

The miR-17-92 binding sites analysis was similar to the codon usage. Here too randomization was done 10,000 taking a random group of genes in the same size of the genes with binding sites for miR-17-92 miRNAs (104), and computing for each distance from the APA site the difference in the number of conserved binding sites for the random group and for all genes. * indicates one-sided p-value < 0.05 for the null hypothesis that for a random group of genes in the same size as the original one, the difference between the number of conserved binding sites between this group and all genes is as good as for the original group or higher.

Analysis of target genes for miR-17-92 cluster miRNAs was done using Gorilla tool [30]. Genes with at least two conserved binding sites for miRNAs in the cluster were defined as the “target set”, and were analyzed in comparison to the background group which was the whole genome.

## Supporting Information

S1 Table Enrichment of different GO categories to target genes of miR-17-92 cluster miRNAs. (PDF)

S2 Table 3’seq results for WI38 cells. RUN_S, RUN_E, RUN_MAX—the location of the APA site which may fluctuate, RUN_MAX is the strongest and was taken into account in our analysis InternalPrim_FLAG—"suspected" due to internal priming to A-rich genomic segments, didn’t include these locations in our analysis.
(XLSX)

S3 Table Table of oligonucleotides for the 3’seq method. (PDF)

S1 Fig Conserved and non-conserved miRNA binding sites around the APA site, for genes with at least 1000 3’ UTR bases from each side of the APA site in the different WI-38 stages **(A)**, or appearing in one cell line and not in the other: **(B)** U2OS not in BJ, **(C)** BJ not in MCF10A, **(D)** WI38 not in BJ and **(E)** WI38 not in MCF10A.
(TIF)

S2 Fig Conserved miRNA binding sites along the 3’UTR in percentage, for all genes with 3’UTR length of at least 1000 3’ UTR bases, with and without APA site in WI38 cells. (TIF)

S3 Fig PhastCons conservation profile around APA sites of genes with at least 1000 bases from each side of the APA site. The genes are divided into those with, or without, conserved miRNA binding sites in the 300 bases 5’ to the APA site.
(TIF)

S4 Fig Conserved and non-conserved miRNA binding sites for genes with APA site and at least 1000 (500 for GSEA GO groups) 3’ UTR bases around it are divided in different groups according to codon usage correlation 0.7 **(A)**, 0.8 **(B)** and top 50 GO groups by correlation according to GSEA **(C)**. * indicates p-value<0.05 for the difference between conserved miRNA binding sites between Pro-Diff. and Pro-Prolif. Genes.
(TIF)

S5 Fig mean fold change (log2 scale) of each miRNA of miR-17-92 cluster in the miRNA array experiment from the control (primary cells) sample. (TIF)
