# Correction: 3'UTR Shortening Potentiates MicroRNA-Based Repression of Pro-differentiation Genes in Proliferating Human Cells

**DOI:** 10.1371/journal.pgen.1006254

**Published:** 2016-08-05

**Authors:** The PLOS Genetics Staff

There is an error in the citation for Dr. Alejandro P. Ugalde. His name should be written Ugalde AP. Please view the correct citation below. The publisher apologizes for the error.

Hoffman Y, Bublik DR, Ugalde AP, Elkon R, Biniashvili T, Agami R, et al. (2016) 3’UTR Shortening Potentiates MicroRNA-Based Repression of Pro-differentiation Genes in Proliferating Human Cells. PLoS Genet 12(2): e1005879. doi:10.1371/journal.pgen.1005879
