# Supplementary material for: 3’UTR Shortening Potentiates MicroRNA-Based Repression of Pro-differentiation Genes in Proliferating Human Cells
Source: PLoS Genet. 2016 Feb 23;12(2):e1005879. doi: 10.1371/journal.pgen.1005879 (PMC4764332; doi:10.1371/journal.pgen.1005879)
Supplement: S1 Table — (PDF) [file pgen.1005879.s001.pdf]

GO term	Description	P-value	FDR q-value	Expected number of overlap
GO:0006357	regulation of transcription from RNA polymerase II promoter	2.65E-16	3.73E-12	60.21
GO:2000113	negative regulation of cellular macromolecule biosynthetic process	3.86E-15	2.71E-11	44.04
GO:0010629	negative regulation of gene expression	1.04E-14	4.89E-11	50.25
GO:0010558	negative regulation of macromolecule biosynthetic process	2.22E-13	7.81E-10	46.95
GO:0009893	positive regulation of metabolic process	4.14E-13	1.17E-09	119.33
GO:0010556	regulation of macromolecule biosynthetic process	5.38E-13	1.26E-09	128.16
GO:0031327	negative regulation of cellular biosynthetic process	1.06E-12	2.14E-09	48.88
GO:0000122	negative regulation of transcription from RNA polymerase II promoter	1.59E-12	2.79E-09	25.11
GO:2000112	regulation of cellular macromolecule biosynthetic process	1.77E-12	2.77E-09	124.55
GO:0009890	negative regulation of biosynthetic process	2.75E-12	3.86E-09	49.65
GO:0019222	regulation of metabolic process	3.11E-12	3.98E-09	216.11
GO:0009889	regulation of biosynthetic process	6.33E-12	7.42E-09	135.74
GO:0051172	negative regulation of nitrogen compound metabolic process	6.78E-12	7.33E-09	49.69
GO:0010468	regulation of gene expression	8.62E-12	8.65E-09	137.03
GO:0060255	regulation of macromolecule metabolic process	1.13E-11	1.06E-08	187.04
GO:0051252	regulation of RNA metabolic process	1.57E-11	1.38E-08	117.15
GO:0010604	positive regulation of macromolecule metabolic process	1.86E-11	1.53E-08	96.46
GO:0045892	negative regulation of transcription, DNA-templated	2.97E-11	2.32E-08	38.85
GO:0010605	negative regulation of macromolecule metabolic process	3.09E-11	2.29E-08	79.28
GO:0080090	regulation of primary metabolic process	4.51E-11	3.17E-08	186.44
GO:0048519	negative regulation of biological process	4.52E-11	3.02E-08	150.01
GO:0032502	developmental process	4.87E-11	3.11E-08	159.09
GO:0031326	regulation of cellular biosynthetic process	4.89E-11	2.99E-08	134.23
GO:0048856	anatomical structure development	5.36E-11	3.14E-08	96.11

GO:0031325	positive regulation of cellular metabolic process	6.63E-11	3.73E-08	97.20
GO:0006351	transcription, DNA-templated	7.12E-11	3.85E-08	77.04
GO:0097659	nucleic acid-templated transcription	7.35E-11	3.83E-08	77.07
GO:0031323	regulation of cellular metabolic process	8.23E-11	4.13E-08	189.28
GO:1903506	regulation of nucleic acid-templated transcription	8.65E-11	4.19E-08	113.47
GO:0051171	regulation of nitrogen compound metabolic process	1.06E-10	4.98E-08	137.10
GO:0006355	regulation of transcription, DNA-templated	1.10E-10	4.99E-08	112.94
GO:2001141	regulation of RNA biosynthetic process	1.41E-10	6.21E-08	114.14
GO:0048523	negative regulation of cellular process	1.45E-10	6.16E-08	137.56
GO:1903507	negative regulation of nucleic acid-templated transcription	1.61E-10	6.66E-08	40.18
GO:0019219	regulation of nucleobase-containing compound metabolic process	2.36E-10	9.49E-08	127.78
GO:1902679	negative regulation of RNA biosynthetic process	3.05E-10	1.19E-07	40.71
GO:0048522	positive regulation of cellular process	4.65E-10	1.77E-07	154.50
GO:0051253	negative regulation of RNA metabolic process	4.95E-10	1.83E-07	41.83
GO:0051254	positive regulation of RNA metabolic process	5.40E-10	1.95E-07	49.93
GO:0032774	RNA biosynthetic process	5.75E-10	2.02E-07	86.75
GO:0048518	positive regulation of biological process	2.20E-09	7.55E-07	179.04
GO:0031324	negative regulation of cellular metabolic process	2.59E-09	8.65E-07	78.05
GO:0006464	cellular protein modification process	3.30E-09	1.08E-06	95.66
GO:0036211	protein modification process	3.30E-09	1.06E-06	95.66
GO:0044260	cellular macromolecule metabolic process	3.84E-09	1.20E-06	218.81
GO:0010557	positive regulation of macromolecule biosynthetic process	5.43E-09	1.66E-06	55.26
GO:0051173	positive regulation of nitrogen compound metabolic process	5.60E-09	1.68E-06	59.93
GO:0009892	negative regulation of metabolic process	5.89E-09	1.72E-06	88.12
GO:0010628	positive regulation of gene expression	7.05E-09	2.02E-06	58.63
GO:0050794	regulation of cellular process	7.56E-09	2.13E-06	317.48
GO:0044767	single-organism developmental process	9.59E-09	2.64E-06	145.98

GO:0007156	homophilic cell adhesion via plasma membrane adhesion molecules	1.14E-08	3.08E-06	5.22
GO:0045935	positive regulation of nucleobase-containing compound metabolic process	1.30E-08	3.44E-06	56.98
GO:0045893	positive regulation of transcription, DNA-templated	1.31E-08	3.41E-06	47.83
GO:1903508	positive regulation of nucleic acid-templated transcription	1.31E-08	3.35E-06	47.83
GO:0045934	negative regulation of nucleobase-containing compound metabolic process	1.35E-08	3.38E-06	46.36
GO:0043412	macromolecule modification	1.41E-08	3.48E-06	101.09
GO:1902680	positive regulation of RNA biosynthetic process	1.43E-08	3.47E-06	48.67
GO:0048731	system development	2.33E-08	5.55E-06	22.76
GO:0043170	macromolecule metabolic process	2.35E-08	5.50E-06	241.81
GO:0007399	nervous system development	2.52E-08	5.80E-06	8.94
GO:0017148	negative regulation of translation	3.44E-08	7.79E-06	3.37
GO:0009891	positive regulation of biosynthetic process	3.58E-08	7.98E-06	60.49
GO:0009059	macromolecule biosynthetic process	7.08E-08	1.56E-05	106.98
GO:0034654	nucleobase-containing compound biosynthetic process	7.64E-08	1.65E-05	95.97
GO:0045944	positive regulation of transcription from RNA polymerase II promoter	8.51E-08	1.81E-05	34.15
GO:0034249	negative regulation of cellular amide metabolic process	8.71E-08	1.83E-05	3.58
GO:0008285	negative regulation of cell proliferation	8.83E-08	1.83E-05	22.37
GO:0007167	enzyme linked receptor protein signaling pathway	9.03E-08	1.84E-05	33.49
GO:0031328	positive regulation of cellular biosynthetic process	1.04E-07	2.08E-05	59.40
GO:0034645	cellular macromolecule biosynthetic process	1.81E-07	3.58E-05	97.31
GO:0019438	aromatic compound biosynthetic process	2.09E-07	4.08E-05	98.39
GO:0048513	animal organ development	3.06E-07	5.90E-05	42.81
GO:0018130	heterocycle biosynthetic process	3.20E-07	6.08E-05	98.22
GO:0050789	regulation of biological process	3.56E-07	6.67E-05	335.19
GO:0098742	cell-cell adhesion via plasma-membrane adhesion molecules	4.83E-07	8.94E-05	6.94

GO:0016070	RNA metabolic process	9.19E-07	1.68E-04	108.67
GO:0065007	biological regulation	1.12E-06	2.02E-04	351.60
GO:1901362	organic cyclic compound biosynthetic process	1.43E-06	2.54E-04	102.46
GO:0048869	cellular developmental process	2.54E-06	4.46E-04	84.51
GO:0044093	positive regulation of molecular function	3.16E-06	5.48E-04	59.86
GO:0044267	cellular protein metabolic process	3.51E-06	6.02E-04	115.54
GO:0007179	transforming growth factor beta receptor signaling pathway	4.09E-06	6.92E-04	4.66
GO:0044271	cellular nitrogen compound biosynthetic process	4.89E-06	8.18E-04	109.09
GO:0032268	regulation of cellular protein metabolic process	6.39E-06	1.06E-03	80.09
GO:0050793	regulation of developmental process	6.92E-06	1.13E-03	70.94
GO:0006468	protein phosphorylation	7.19E-06	1.16E-03	31.14
GO:0045595	regulation of cell differentiation	7.69E-06	1.23E-03	49.79
GO:0051246	regulation of protein metabolic process	8.14E-06	1.29E-03	85.63
GO:0051247	positive regulation of protein metabolic process	9.05E-06	1.41E-03	52.42
GO:0032270	positive regulation of cellular protein metabolic process	9.67E-06	1.49E-03	49.30
GO:0043085	positive regulation of catalytic activity	9.74E-06	1.49E-03	50.91
GO:2000026	regulation of multicellular organismal development	1.05E-05	1.59E-03	53.44
GO:0010646	regulation of cell communication	1.10E-05	1.65E-03	100.08
GO:0035194	posttranscriptional gene silencing by RNA	1.13E-05	1.68E-03	0.35
GO:0060211	regulation of nuclear-transcribed mRNA poly(A) tail shortening	1.13E-05	1.66E-03	0.35
GO:0060213	positive regulation of nuclear-transcribed mRNA poly(A) tail shortening	1.13E-05	1.64E-03	0.35
GO:0030154	cell differentiation	1.65E-05	2.36E-03	58.17
GO:0031401	positive regulation of protein modification process	1.79E-05	2.55E-03	40.61
GO:0016441	posttranscriptional gene silencing	2.02E-05	2.84E-03	0.39
GO:0044708	single-organism behavior	2.22E-05	3.09E-03	13.75
GO:0007169	transmembrane receptor protein tyrosine kinase signaling pathway	2.37E-05	3.26E-03	26.44
GO:0051239	regulation of multicellular organismal process	2.76E-05	3.76E-03	85.24

GO:0010941	regulation of cell death	3.34E-05	4.51E-03	51.86
GO:0043124	negative regulation of I-kappaB kinase/NF-kappaB signaling	3.72E-05	4.97E-03	1.68
GO:0042325	regulation of phosphorylation	4.20E-05	5.57E-03	49.76
GO:0010990	regulation of SMAD protein complex assembly	4.29E-05	5.63E-03	0.11
GO:0010991	negative regulation of SMAD protein complex assembly	4.29E-05	5.58E-03	0.11
GO:0060284	regulation of cell development	4.86E-05	6.27E-03	27.95
GO:0035556	intracellular signal transduction	5.04E-05	6.44E-03	59.93
GO:0006417	regulation of translation	5.30E-05	6.71E-03	9.82
GO:0042327	positive regulation of phosphorylation	5.40E-05	6.77E-03	34.96
GO:0044238	primary metabolic process	5.73E-05	7.13E-03	282.38
GO:0043547	positive regulation of GTPase activity	5.82E-05	7.17E-03	16.52
GO:0090304	nucleic acid metabolic process	5.82E-05	7.12E-03	125.01
GO:0023051	regulation of signaling	6.82E-05	8.26E-03	99.30
GO:0016579	protein deubiquitination	6.96E-05	8.36E-03	3.16
GO:0009653	anatomical structure morphogenesis	7.60E-05	9.05E-03	46.57
GO:0043067	regulation of programmed cell death	7.65E-05	9.04E-03	49.02
GO:0042127	regulation of cell proliferation	7.88E-05	9.23E-03	50.70
GO:0010608	posttranscriptional regulation of gene expression	7.97E-05	9.25E-03	14.03
GO:1900151	regulation of nuclear-transcribed mRNA catabolic process, deadenylation-dependent decay	8.02E-05	9.23E-03	0.49
GO:1900153	positive regulation of nuclear-transcribed mRNA catabolic process, deadenylation-dependent decay	8.02E-05	9.16E-03	0.49
GO:0043087	regulation of GTPase activity	8.36E-05	9.47E-03	18.23
GO:0070536	protein K63-linked deubiquitination	8.36E-05	9.40E-03	0.77
GO:0009966	regulation of signal transduction	8.94E-05	9.97E-03	86.65
GO:0048286	lung alveolus development	8.95E-05	9.91E-03	1.47
GO:0051726	regulation of cell cycle	9.88E-05	1.08E-02	33.38
GO:0042981	regulation of apoptotic process	1.02E-04	1.11E-02	48.64

GO:0016310	phosphorylation	1.04E-04	1.12E-02	37.38
GO:0050767	regulation of neurogenesis	1.07E-04	1.15E-02	22.09
GO:0016568	chromatin modification	1.12E-04	1.19E-02	17.08
GO:0021953	central nervous system neuron differentiation	1.17E-04	1.24E-02	2.84
GO:0001934	positive regulation of protein phosphorylation	1.26E-04	1.33E-02	33.70
GO:0007178	transmembrane receptor protein serine/threonine kinase signaling pathway	1.29E-04	1.34E-02	7.26
GO:0007610	behavior	1.34E-04	1.39E-02	17.95
GO:0065009	regulation of molecular function	1.59E-04	1.64E-02	93.13
GO:0048015	phosphatidylinositol-mediated signaling	1.60E-04	1.63E-02	4.49
GO:0007165	signal transduction	1.62E-04	1.64E-02	148.68
GO:0031442	positive regulation of mRNA 3'-end processing	1.65E-04	1.66E-02	0.56
GO:0044707	single-multicellular organism process	1.67E-04	1.67E-02	89.70
GO:0007632	visual behavior	1.73E-04	1.71E-02	2.03
GO:0010562	positive regulation of phosphorus metabolic process	1.83E-04	1.79E-02	38.96
GO:0045937	positive regulation of phosphate metabolic process	1.83E-04	1.78E-02	38.96
GO:0071704	organic substance metabolic process	1.87E-04	1.82E-02	291.95
GO:0048017	inositol lipid-mediated signaling	2.04E-04	1.97E-02	4.59
GO:0002064	epithelial cell development	2.13E-04	2.04E-02	4.07
GO:0038095	Fc-epsilon receptor signaling pathway	2.17E-04	2.06E-02	10.10
GO:0034248	regulation of cellular amide metabolic process	2.19E-04	2.07E-02	10.76
GO:0060009	Sertoli cell development	2.27E-04	2.13E-02	0.60
GO:0044237	cellular metabolic process	2.31E-04	2.15E-02	277.50
GO:0017015	regulation of transforming growth factor beta receptor signaling pathway	2.35E-04	2.17E-02	3.58
GO:1903844	regulation of cellular response to transforming growth factor beta stimulus	2.35E-04	2.15E-02	3.58
GO:0003279	cardiac septum development	2.38E-04	2.17E-02	1.68

GO:0031399	regulation of protein modification process	2.39E-04	2.17E-02	59.22
GO:0042992	negative regulation of transcription factor import into nucleus	2.59E-04	2.33E-02	1.30
GO:0035871	protein K11-linked deubiquitination	2.65E-04	2.38E-02	0.35
GO:0051347	positive regulation of transferase activity	2.74E-04	2.43E-02	23.84
GO:0051603	proteolysis involved in cellular protein catabolic process	2.95E-04	2.61E-02	14.41
GO:0070647	protein modification by small protein conjugation or removal	2.97E-04	2.61E-02	28.54
GO:0007507	heart development	3.02E-04	2.63E-02	6.52
GO:0044249	cellular biosynthetic process	3.05E-04	2.65E-02	135.49
GO:0035051	cardiocyte differentiation	3.08E-04	2.65E-02	1.33
GO:0042787	protein ubiquitination involved in ubiquitin-dependent protein catabolic process	3.08E-04	2.64E-02	3.68
GO:0043068	positive regulation of programmed cell death	3.09E-04	2.63E-02	20.23
GO:0019220	regulation of phosphate metabolic process	3.11E-04	2.63E-02	57.16
GO:1901576	organic substance biosynthetic process	3.25E-04	2.74E-02	140.30
GO:0070646	protein modification by small protein removal	3.37E-04	2.82E-02	3.72
GO:0051960	regulation of nervous system development	3.41E-04	2.84E-02	24.86
GO:0007219	Notch signaling pathway	3.66E-04	3.03E-02	5.44
GO:0033674	positive regulation of kinase activity	3.85E-04	3.17E-02	21.21
GO:0007611	learning or memory	3.97E-04	3.25E-02	7.92
GO:0051174	regulation of phosphorus metabolic process	3.99E-04	3.24E-02	57.61
GO:0061014	positive regulation of mRNA catabolic process	4.02E-04	3.25E-02	0.67
GO:0034773	histone H4-K20 trimethylation	4.07E-04	3.27E-02	0.18
GO:0001932	regulation of protein phosphorylation	4.10E-04	3.28E-02	46.74
GO:0019538	protein metabolic process	4.41E-04	3.50E-02	138.33
GO:0030182	neuron differentiation	4.43E-04	3.50E-02	7.99
GO:0010001	glial cell differentiation	4.69E-04	3.68E-02	2.31
GO:0008542	visual learning	4.79E-04	3.74E-02	1.86
GO:0051345	positive regulation of hydrolase activity	4.93E-04	3.82E-02	27.63

GO:0071496	cellular response to external stimulus	5.09E-04	3.93E-02	8.73
GO:0043549	regulation of kinase activity	5.10E-04	3.91E-02	30.82
GO:0048145	regulation of fibroblast proliferation	5.20E-04	3.97E-02	2.84
GO:0051094	positive regulation of developmental process	5.21E-04	3.96E-02	38.08
GO:0014911	positive regulation of smooth muscle cell migration	5.24E-04	3.96E-02	1.05
GO:0043065	positive regulation of apoptotic process	5.26E-04	3.95E-02	20.06
GO:0009628	response to abiotic stimulus	5.54E-04	4.14E-02	38.99
GO:1902531	regulation of intracellular signal transduction	5.71E-04	4.25E-02	54.88
GO:0032501	multicellular organismal process	5.77E-04	4.27E-02	111.44
GO:0051128	regulation of cellular component organization	6.27E-04	4.61E-02	71.43
GO:0050678	regulation of epithelial cell proliferation	6.27E-04	4.59E-02	9.54
GO:0048708	astrocyte differentiation	6.65E-04	4.84E-02	0.74
GO:0007173	epidermal growth factor receptor signaling pathway	6.71E-04	4.86E-02	10.94
GO:0090288	negative regulation of cellular response to growth factor stimulus	6.99E-04	5.03E-02	4.59
GO:0045860	positive regulation of protein kinase activity	7.20E-04	5.17E-02	20.41
GO:0016197	endosomal transport	7.37E-04	5.26E-02	7.05
GO:0003281	ventricular septum development	7.53E-04	5.35E-02	1.12
GO:0033554	cellular response to stress	7.59E-04	5.36E-02	48.64
GO:0007033	vacuole organization	7.65E-04	5.37E-02	2.98
GO:2000766	negative regulation of cytoplasmic translation	7.92E-04	5.54E-02	0.21
GO:0045974	regulation of translation, ncRNA-mediated	7.92E-04	5.51E-02	0.21
GO:0040033	negative regulation of translation, ncRNA-mediated	7.92E-04	5.49E-02	0.21
GO:0036124	histone H3-K9 trimethylation	7.92E-04	5.46E-02	0.21
GO:0035278	miRNA mediated inhibition of translation	7.92E-04	5.43E-02	0.21
GO:0050890	cognition	7.96E-04	5.43E-02	9.05
GO:0038127	ERBB signaling pathway	7.99E-04	5.42E-02	11.08
GO:0009058	biosynthetic process	8.06E-04	5.45E-02	142.82

GO:0061013	regulation of mRNA catabolic process	8.35E-04	5.62E-02	0.77
GO:0007411	axon guidance	8.41E-04	5.63E-02	19.08
GO:0010942	positive regulation of cell death	8.48E-04	5.65E-02	21.35
GO:0097485	neuron projection guidance	8.68E-04	5.75E-02	19.11
GO:0090207	regulation of triglyceride metabolic process	8.94E-04	5.90E-02	1.16
GO:0030512	negative regulation of transforming growth factor beta receptor signaling pathway	8.95E-04	5.88E-02	2.52
GO:1903845	negative regulation of cellular response to transforming growth factor beta stimulus	8.95E-04	5.85E-02	2.52
GO:0006796	phosphate-containing compound metabolic process	9.19E-04	5.98E-02	62.66
GO:0006511	ubiquitin-dependent protein catabolic process	9.48E-04	6.14E-02	12.62
GO:0051248	negative regulation of protein metabolic process	9.81E-04	6.32E-02	35.80
GO:0045670	regulation of osteoclast differentiation	9.98E-04	6.40E-02	2.07
