# Supplementary material for: 3’UTR Shortening Potentiates MicroRNA-Based Repression of Pro-differentiation Genes in Proliferating Human Cells
Source: PLoS Genet. 2016 Feb 23;12(2):e1005879. doi: 10.1371/journal.pgen.1005879 (PMC4764332; doi:10.1371/journal.pgen.1005879)
Supplement: S3 Table — (PDF) [file pgen.1005879.s003.pdf]

Oligonucleotides for 3'Seq

| **Name** | **Sequence (5'-3')** |
|---|---|
| P5 | AATGATACGGCGACCACCGA |
| P7 | CAAGCAGAAGACGGCATACGAGAT |
| P7t25VN | CAAGCAGAAGACGGCATACGAGATTTTTTTTTTTTTTTTTTTTTTTTTVN |
| 3seq_RT1 | TTTTTTTTTTTTTTTTTTTGcagtAGATCGGAAGAGCGCTACACGACGCTCTTCCGATCTactgCTTTTTTVN |
| 3seq_RT2 | TTTTTTTTTTTTTTTTTTTGatcgAGATCGGAAGAGCGCTACACGACGCTCTTCCGATCTcgatCTTTTTTVN |
| 3seq_RT3 | TTTTTTTTTTTTTTTTTTTGtgacAGATCGGAAGAGCGCTACACGACGCTCTTCCGATCTgtcaCTTTTTTVN |
| 3seq_RT4 | TTTTTTTTTTTTTTTTTTTCgctaAGATCGGAAGAGCGCTACACGACGCTCTTCCGATCTtagcGTTTTTTVN |
| 3seq_RT5 | TTTTTTTTTTTTTTTTTTTGagctAGATCGGAAGAGCGCTACACGACGCTCTTCCGATCTagctCTTTTTTVN |
| 3seq_RT6 | TTTTTTTTTTTTTTTTTTTGctagAGATCGGAAGAGCGCTACACGACGCTCTTCCGATCTctagCTTTTTTVN |
| 3seq_RT7 | TTTTTTTTTTTTTTTTTTTCgatcAGATCGGAAGAGCGCTACACGACGCTCTTCCGATCTgatcGTTTTTTVN |
| 3seq_RT8 | TTTTTTTTTTTTTTTTTTTCtcgaAGATCGGAAGAGCGCTACACGACGCTCTTCCGATCTtcgaGTTTTTTVN |
| 3seq_RT9 | TTTTTTTTTTTTTTTTTTTGcgatAGATCGGAAGAGCGCTACACGACGCTCTTCCGATCTatcgCTTTTTTVN |
| 3seq_RT10 | TTTTTTTTTTTTTTTTTTTCactgAGATCGGAAGAGCGCTACACGACGCTCTTCCGATCTcagtGTTTTTTVN |
| 3seq_RT11 | TTTTTTTTTTTTTTTTTTTCgtgcAGATCGGAAGAGCGCTACACGACGCTCTTCCGATCTgcacGTTTTTTVN |
| 3seq_RT12 | TTTTTTTTTTTTTTTTTTTCtacaAGATCGGAAGAGCGCTACACGACGCTCTTCCGATCTtgtaGTTTTTTVN |
| Illumina_Truseq | AATGATACGGCGACCACCGAGATCTACACTCTTTCCCTACACGACGCTCTTCCGATCT |
