# Supplementary figures and images for: 3’UTR Shortening Potentiates MicroRNA-Based Repression of Pro-differentiation Genes in Proliferating Human Cells
Source: PLoS Genet. 2016 Feb 23;12(2):e1005879. doi: 10.1371/journal.pgen.1005879 (PMC4764332; doi:10.1371/journal.pgen.1005879)

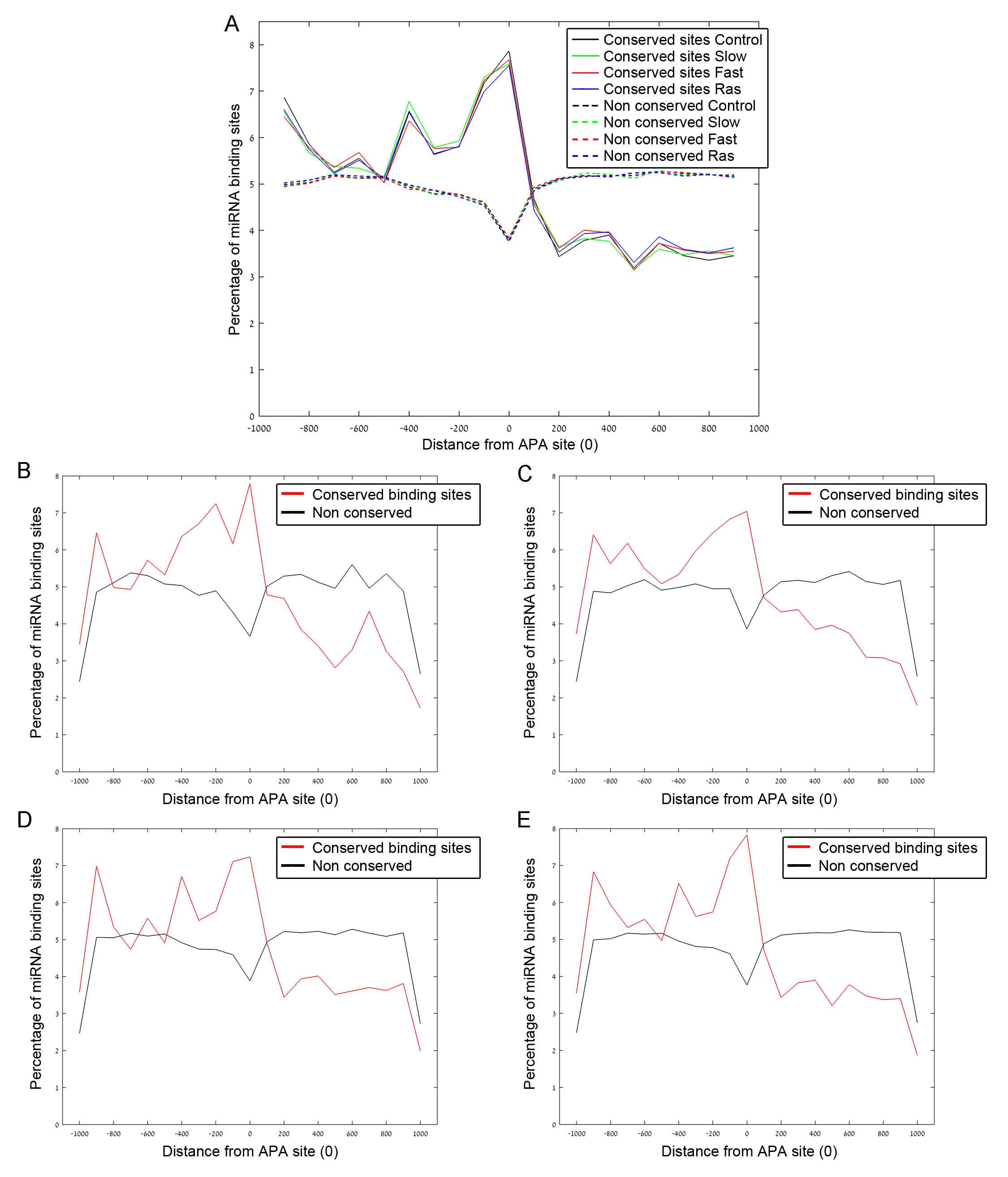

Supplement: S1 Fig — Conserved and non-conserved miRNA binding sites around the APA site, for genes with at least 1000 3’ UTR bases from each side of the APA site in the different WI-38 stages (A), or appearing in one cell line and not in the other: (B) U2OS not in BJ, (C) BJ not in MCF10A, (D) WI38 not in BJ and (E) WI38 not in MCF10A. (TIF) [file pgen.1005879.s004.tif]

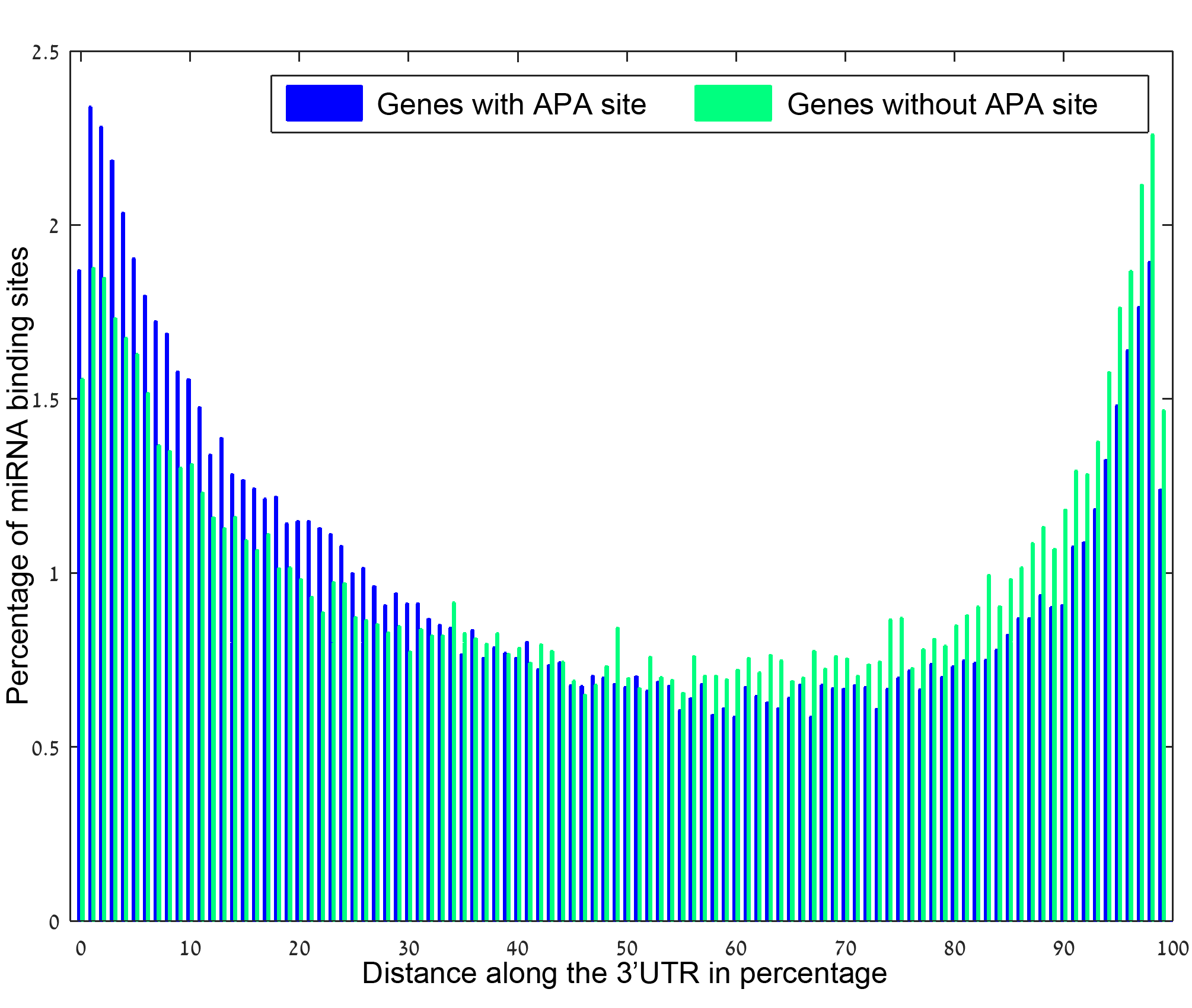

Supplement: S2 Fig — (TIF) [file pgen.1005879.s005.tif]

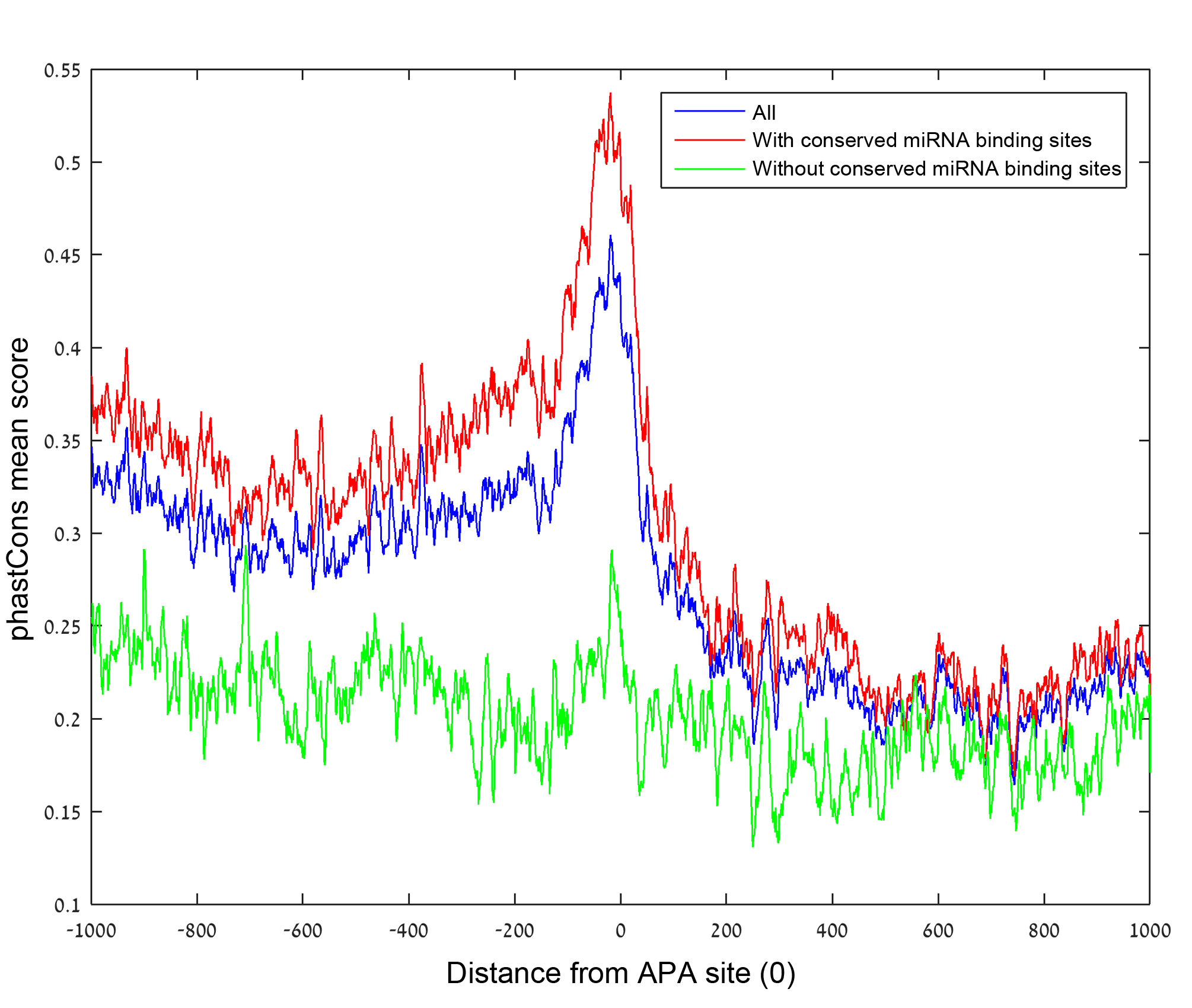

Supplement: S3 Fig — The genes are divided into those with, or without, conserved miRNA binding sites in the 300 bases 5’ to the APA site. (TIF) [file pgen.1005879.s006.tif]

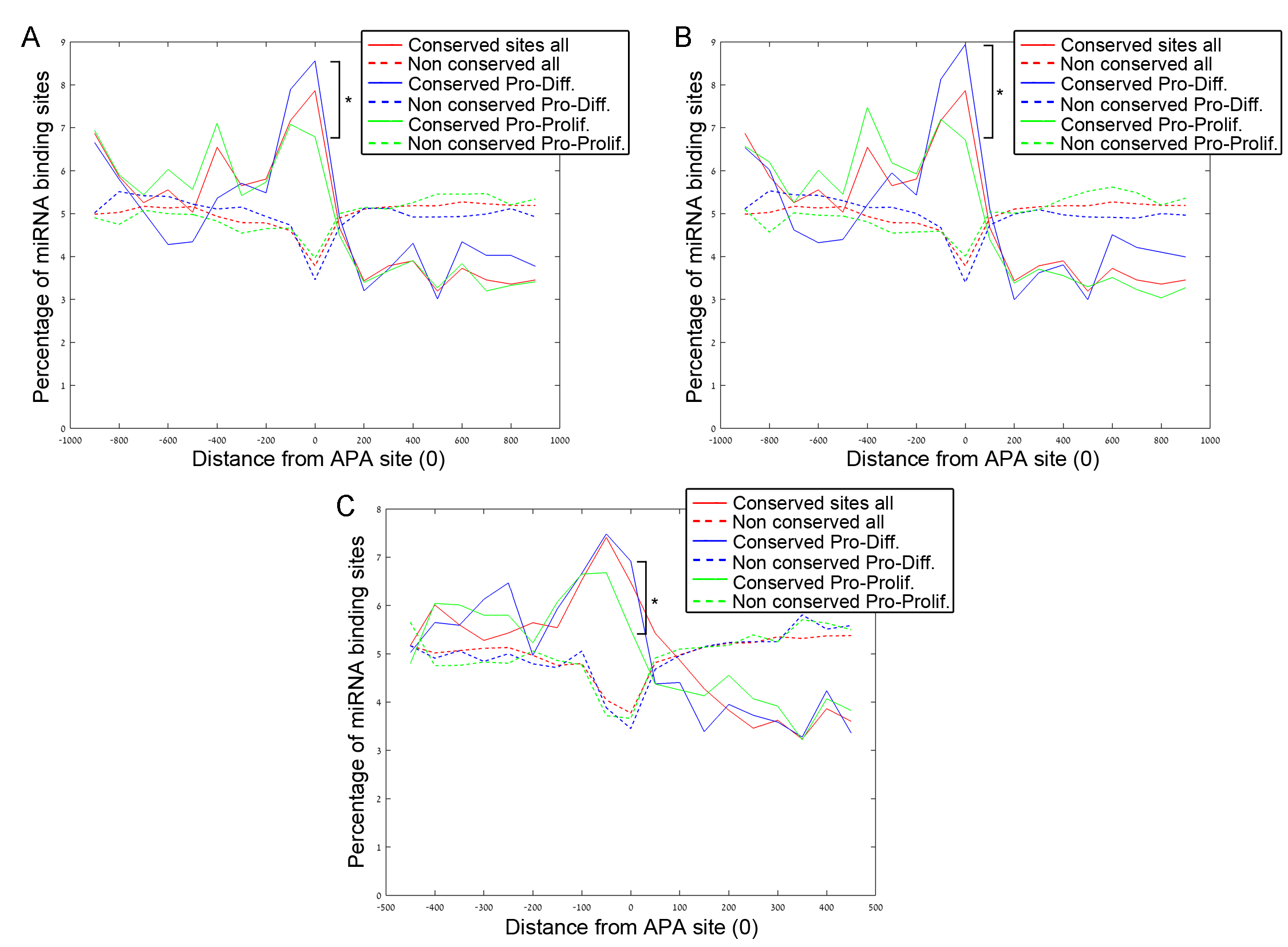

Supplement: S4 Fig — Conserved and non-conserved miRNA binding sites for genes with APA site and at least 1000 (500 for GSEA GO groups) 3’ UTR bases around it are divided in different groups according to codon usage correlation 0.7 (A), 0.8 (B) and top 50 GO groups by correlation according to GSEA (C). * indicates p-value<0.05 for the difference between conserved miRNA binding sites between Pro-Diff. and Pro-Prolif. Genes. (TIF) [file pgen.1005879.s007.tif]

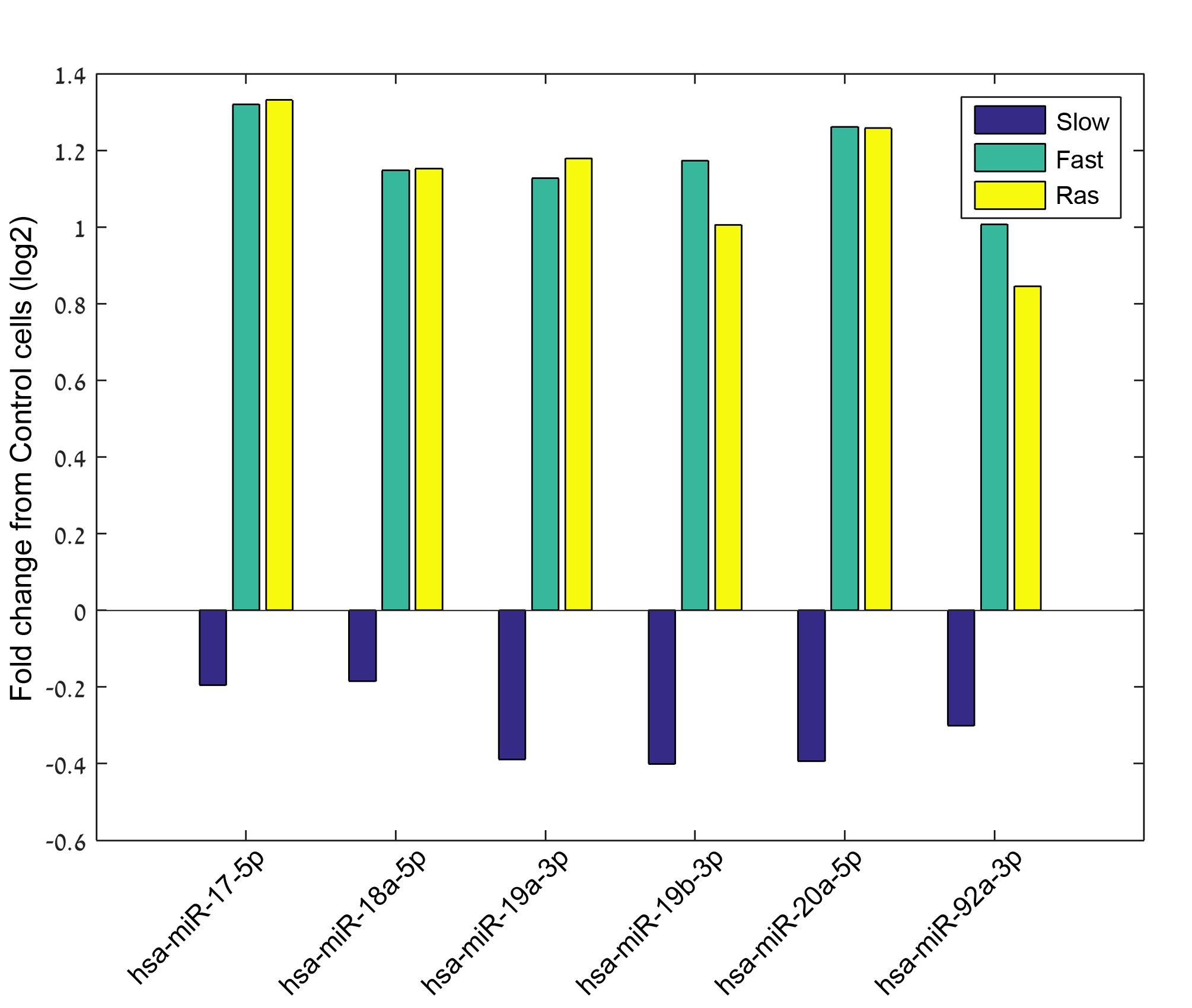

Supplement: S5 Fig — (TIF) [file pgen.1005879.s008.tif]
